# A review of the status of sarcopenia in elderly hospitalized patients

**DOI:** 10.1097/MD.0000000000043365

**Published:** 2025-07-11

**Authors:** Xuejiao Xian, Jianbin Zhang, Hongjun Yang

**Affiliations:** a Department of General Surgery, Chuxiong Yi Autonomous Prefecture People’s Hospital, Lucheng Town, Chuxiong City, Chuxiong Yi Autonomous Prefecture, Yunnan Province, China; b Department of Emergency, Chuxiong Yi Autonomous Prefecture People’s Hospital, Lucheng Town, Chuxiong City, Chuxiong Yi Autonomous Prefecture, Yunnan Province, China; c Department of General Surgery, The First Affiliated Hospital of Kunming Medical University, Wuhua District, Kunming City, Yunnan Province, China.

**Keywords:** hospitalized patients, older adults, review, sarcopenia

## Abstract

The prevalence of sarcopenia among hospitalized patients has shown a progressive increase across multiple regions in recent years. This review systematically examines the diagnostic criteria, current research trends, and therapeutic approaches for sarcopenia, aiming to enhance both clinical practitioners’ and patients’ comprehension of this condition. Furthermore, it seeks to facilitate the implementation of tailored and evidence-based treatment strategies for hospitalized sarcopenia patients, thereby optimizing patient care and safety outcomes.

## 1. Introduction

Sarcopenia, derived from the Greek roots “sarx” (flesh) and “penia” (loss), is a geriatric syndrome first formally described by Rosenberg in 1989 and subsequently defined by the European Sarcopenia Working Group in 2010 as an age-related condition characterized by progressive loss of skeletal muscle mass, diminished muscle strength, and declining physical performance.^[1–3]^ The pathophysiological progression of sarcopenia typically manifests as gradual deterioration of mobility and physical function secondary to musculoskeletal degeneration.^[1,2]^ Sarcopenia is now internationally recognized as a health risk for older adults, and is associated with increased adverse events such as diabetes, risk of falls and hospitalization, disability, psychiatric problems, and mortality.^[4]^

Reports from both domestic and international sources indicate that the prevalence of sarcopenia ranges from 5% to 13% among individuals aged 60 to 70, and from 11% to 50% in those aged 80 and above.^[5,6]^ There are also reports that the prevalence of sarcopenia ranges from 3.9% to 98.5%.^[7–12]^ In recent years, as the State has increased its efforts to support medical care for the whole population, and as the medical insurance system has been further improved, and people’s awareness of health care has been gradually strengthened, the number of in-patient admissions to hospitals has increased to a certain extent compared with that of previous years, and at the same time the number of in-patient admissions with sarcopenia has also increased. The occurrence of sarcopenia in elderly hospitalized patients is associated with major adverse outcomes, such as high rates of re-hospitalization, prolonged hospital stay, and increased risk of death. However, it is worrying that physical exercise and nutritional support are still the conventional treatments for sarcopenia. In the field of drug therapy, although many interventions have been proposed to alleviate muscle aging, none of them has been successfully translated into a definitive treatment for sarcopenia. This article will discuss the diagnostic standardization of sarcopenia in hospitalized patients, future research directions, and related treatment options. In this paper, we will focus on the diagnostic standardization of sarcopenia in hospitalized patients, future research direction and related treatment options.

## 2. Methods

PubMed database and Google Scholar were used from their inception up to February 2, 2025 to search published articles related to sarcopenia in elderly hospitalized patients, using the following key search terms: (“elderly hospitalized patients” OR “older adult hospitalized patients” OR “elderly inpatients” OR “older adults inpatients” OR “hospitalized elderly” OR “hospitalized older adults”) AND “sarcopenia.” Both forward and backward citation searches on the selected references were applied to ensure the inclusion of all relevant studies. The present review includes articles published in English, adults aged ≥ 60 years.

### 2.1. Diagnosis of sarcopenia

#### 2.1.1. Diagnostic criteria

**Figure 1.**
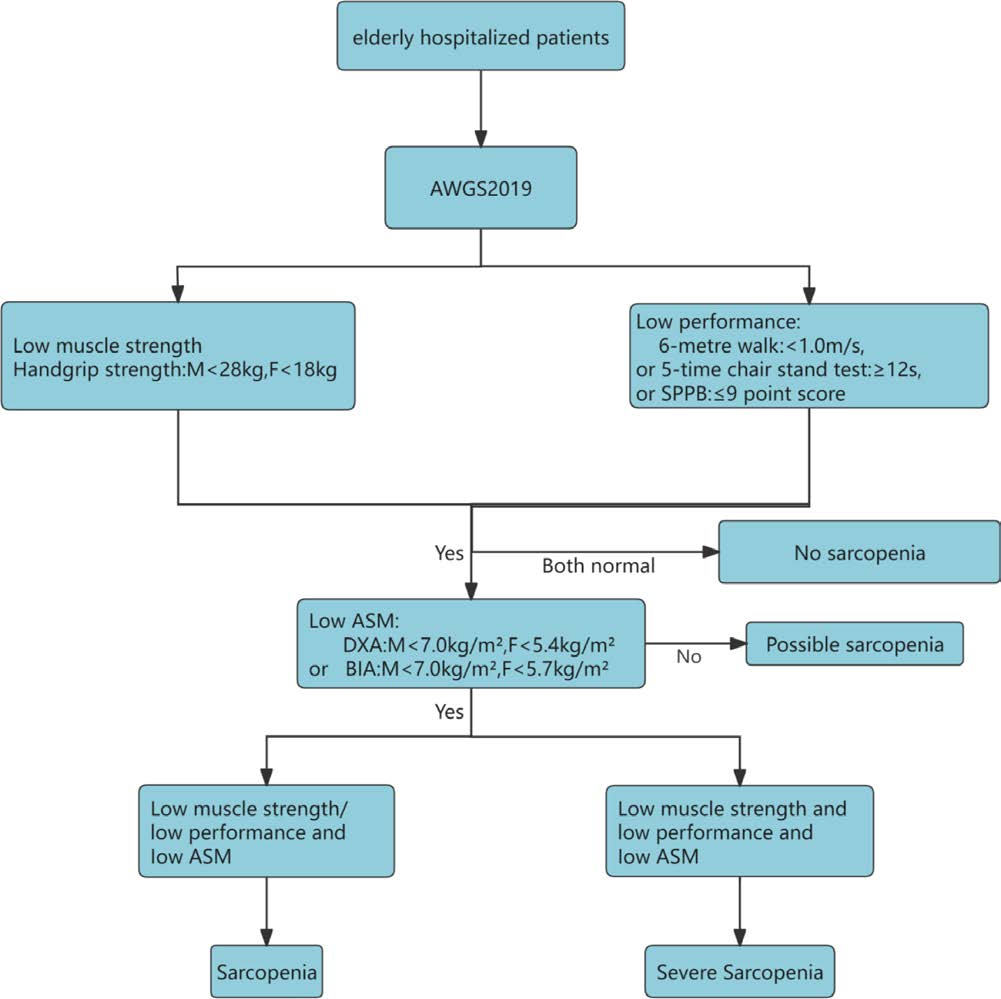
Sarcopenia diagnostic flowchart.

Nowadays, the risk factors that we can consider for the diagnosis and determination of sarcopenia include somatic function, muscle mass, and muscle strength. In view of the differences in the criteria for determining sarcopenia both at home and abroad, in accordance with the recommendations of the Expert Consensus on the Diagnosis and Treatment of Sarcopenia in the Elderly in China (2021), which have been recognized by everyone, Chinese patients with sarcopenia should be diagnosed by adopting the diagnostic reference values of the Asian Working Group on Sarcopenia 2019, that is (1) muscle mass: skeletal muscle mass of limbs/height as measured by dual-energy X-ray absorptiometry^2^ (kg/m^2^). Male ≤ 7.0, female ≤ 5.4 or body composition assessment (BIA) measurement of skeletal muscle mass/height^2^ (kg/m^2^): male ≤ 7.0, female ≤ 5.7, (2) muscle strength is mainly considered to be the grip strength: male <28 kg, female <18 kg, (3) with or without reduction of physical mobility: step speed < 1.0 m/s or 5 sits ≥ 12 seconds or Simple Physical Condition Scale or ≤ 9 points on the Simple Physical Fitness Scale^[13]^ (as shown in Fig. 1).

#### 2.1.2. Assessment tools

##### 2.1.2.1. Muscle mass

Muscle mass is often demonstrated using conventional computed tomography and magnetic resonance imaging, which are the most authoritative standards for estimating muscle mass, and dual-energy X-ray absorptiometry is the preferred alternative method for data analysis and application in practice.^[1]^ Dual-energy X-ray absorptiometry is primarily considered for data analysis and use in clinical practice to differentiate between fat, bone mineral, and lean tissue. The advantage of this body for a full scan is that it allows the patient to be under the lowest radiation. But the biggest disadvantage is that the equipment is expensive, requires a lot of space and is more difficult to handle, which may somewhat affect its use in large-scale epidemiologic analysis explorations.^[14]^ The BIA assessment yields the volume of fat and muscle. The equipment required for this measurement is inexpensive, requires little space, is relatively easy to handle, is simple to perform, can be used multiple times, and is particularly suitable for bedridden patients, and can be promoted for use in hospital outpatient clinics. The research use of the BIA measurement technique has been operated under relatively standardized and fairly regulated conditions for more than 10 years,^[15]^ and it is recognized that the results of BIA are not consistent with the results of BIA under relatively standardized and fairly regulated conditions. We have recognized the strong association between BIA results and magnetic resonance imaging predictions in relatively standardized and well-regulated conditions.^[14]^ Predictive equations have been analyzed in adults of all ages and ethnicities, and it has been found that the best alternative to BIA is probably dual-energy X-ray absorptiometry, which is equally portable.

##### 2.1.2.2. Muscle strength

There are relatively few validated techniques for muscle strength, and we usually think of grip strength as a physical manifestation of overall body muscle strength and total physical performance in the general population, especially in older age groups,^[16]^ poor grip strength values are a clinical manifestation of poor physical mobility, and it is also a better predictor of clinical outcome than low muscle mass.^[17]^ There is also a linear relationship between baseline grip strength and unintentional disability in clinical practice.^[18]^

##### 2.1.2.3. Somatic functions

**Figure 2.**
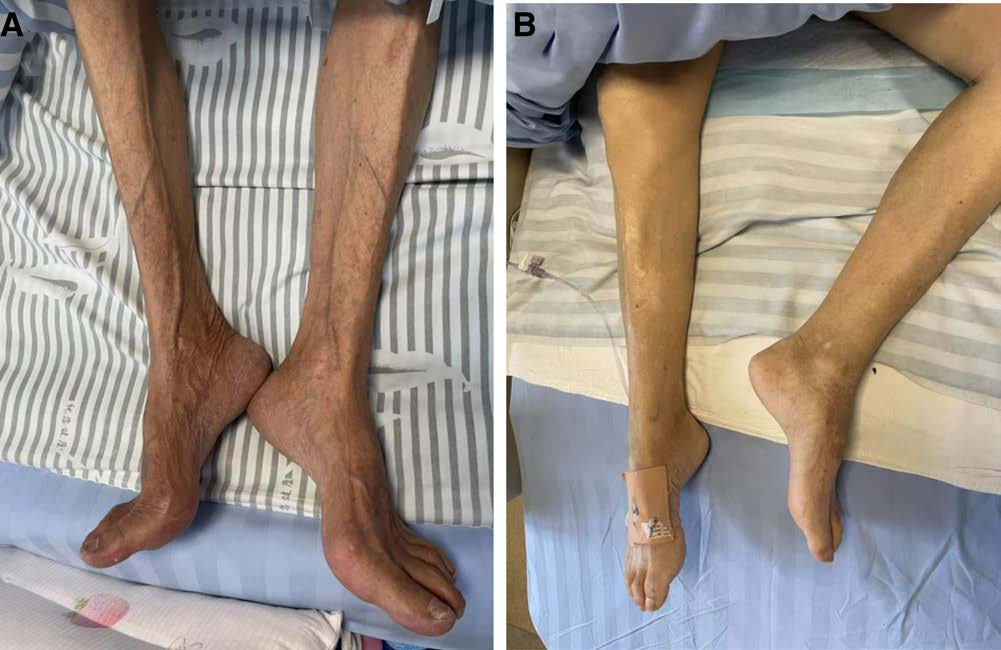
Comparison between sarcopenia patients and non-sarcopenia patients. (A) The calf muscle of a 67-year-old sarcopenia patient with low muscle strength and low performance and low ASM, and (B) the calf muscle of a 67-year-old non-sarcopenia patient.

Normalization of somatic function is usually determined by relying on a variety of physical fitness testing modalities: including the 6-minute walk test, the Brief Physical Status Scale, the Get-Up-Walk Timing Test, and the Stair Climbing Strength Test.^[19]^ Of these testing modalities, pace is the most convenient, easy to administer, quickest, and lowest risk method for subjects with sarcopenia. Pace is associated with early detection of the poor prognosis associated with sarcopenia and is strongly associated with a long and healthy life.^[20]^ The Brief Physical Status Scale is a comprehensive measure of physical performance and is a standardized measure for research and clinical practice. The Stand-Walk Timed Test requires the subject to start the test in a seated position, and at the beginning of the test the subject is required to stand from a stool, walk a short distance, and then return to the stool position and sit down again. Therefore, it can be used as a measure of dynamic balance, where the subject’s balance function can be observed and can be scored on a five-point scale,^[21]^ which is currently less used and is not recommended for generalization. Figure 2 is a comparison of calf images of hospitalized elderly patients with sarcopenia and non-hospitalized elderly patients with sarcopenia.

## 3. Discussions

Depending on the cause of the disease, sarcopenia can be classified as either “primary or “secondary.”^[22,23]^ The aging of the human body is considered to be the most important and probably the only risk for the development of primary sarcopenia, a conclusion that is largely agreed upon, which is why we also refer to primary sarcopenia as “sarcopenia that is strongly associated with the aging of the human body.” Therefore, we also refer to “primary sarcopenia” as “sarcopenia that is strongly associated with aging in humans.” Secondary sarcopenia, as opposed to primary sarcopenia, can be caused by a combination of 1 or more other modifying physical conditions, such as too little physical activity or too much exercise (which is activity-related). For example, people have too little physical activity or too little exercise (this is activity-related sarcopenia), people may suffer from advanced malignant tumors or other very serious diseases, resulting in the failure of various important internal organs (this is disease-related sarcopenia), and due to the lack of knowledge and awareness of nutritional aspects, which may lead to the imbalance of the intake of various types of food and nutrients, resulting in the loss of energy in the body. The body’s energy level is insufficient and protein level is low (nutrition-related sarcopenia) due to a lack of knowledge and awareness of nutritional issues. However, in clinical practice, we have great difficulty in determining whether a patient has primary or secondary sarcopenia, as the patient may have a variety of conditions, especially in the elderly, and we may consider sarcopenia to be a multifaceted geriatric syndrome. The following are the mechanisms discovered so far for the development of sarcopenia:

### 3.1. Aging

It is entirely possible that the incidence of sarcopenia increases significantly with age, and the close relationship between them has been recognized by many studies, both national and international, for many years now. First of all, aging disrupts the balance of skeletal muscle, and it seems that aging may also lead to the unevenness of muscle anabolic proteins, as well as the breakdown of their metabolic channels, which may lead to the complete loss of skeletal muscle.^[24]^ Although national and international studies have confirmed that the development of sarcopenia is also closely related to the gradual increase in age.^[24]^ However, the exact mechanisms and pathways involved in the development of sarcopenia are not yet fully understood. Whether the primary cause is directly related to age-associated physiological changes remains uncertain and requires validation through extensive research data.

Secondly, the main hallmark of aging is damage at the cellular level, that is, it will reflect an unfixed genome, telomeres that may be worn out, epigenetic alterations as well, or is it the case that proteins may appear less stable, or even begin to fluctuate, etc.^[25,26]^ To date, the evidence for a genetic basis as a cause of sarcopenia remains low, although it is beginning to be studied. However, a large number of studies are still looking at genetic influences on sarcopenia based only on skeletal muscle mass or lean body mass.

### 3.2. Cytokine imbalances and insulin resistance

Several studies have shown that one of the most important reasons for the occurrence of sarcopenia in the elderly population is most likely the fact that inflammatory response cytokines in the body exceed normal norms. A combination of facilitated catabolic signaling mediated through pro-inflammatory cytokines such as tumor necrosis factor alpha and interleukin 6.^[27]^ Elevated levels of tumor necrosis factor alpha and interleukin 6 have been shown to be present in skeletal muscle of the elderly. Fairly high masses of interleukin 6, tumor necrosis factor alpha, interleukin 1 beta and interferon gamma are very likely to utilize the attraction of nuclear factor kB and adenine nucleoside triphosphate-ubiquitin to achieve catabolism of proteins, however, with the gradual loss of muscle mass, the risk of a further decrease in muscle strength and an accelerated catabolism of muscle proteins is increased.^[28]^

Insulin resistance and sarcopenia are closely related to each other, as we all know, the largest glycogen storage mechanism within the human body is skeletal muscle, if muscle tissue declines, the storage capacity diminishes, at the same time, too much blood glucose stored in the body can not be converted to glycogen by insulin, resulting in insulin resistance associated with aging.^[29]^ The reduced glucose uptake by peripheral tissues (primarily skeletal muscle) associated with aging is not due to impaired insulin binding, but to a defect in the post-receptor intracellular insulin signaling pathway.^[29,30]^ This defect has not been fully elucidated; however, the number of insulin-stimulated glucose transporter units decreases with age. Thus, fewer glucose transporter proteins 4 and/or post-receptor defects in the insulin signaling cascade response lead to insulin resistance.^[31]^

### 3.3. Inadequate nutritional intake and reduced physical activity

The amount of protein in muscle fibers determines whether human muscle fibers shrink or enlarge. More than 80% of the muscle in the human body is composed of protein,^[32]^ so we can assume that the storage mechanism for protein is the skeletal muscle and that the amount of muscle in the human body depends on the amount of protein, which is under-supplemented in the majority of the population, with malabsorption occurring in the elderly. Theoretically, phosphorylation of mammalian target of rapamycin in myocytes is induced when muscle degradation is increased and synthesis is inhibited.^[33]^ The mammalian target of rapamycin complex 1 signaling pathway is a major regulator of protein metabolism. Mammalian target of rapamycin complex 1 regulates protein synthesis by integrating many intracellular signals for degradation.^[34]^ As mentioned above, one of the reasons why sarcopenia occurs is reduced muscle synthesis; insulin-like growth factor-1 activates the intracellular signaling pathway of phosphatidylinositol 3-kinase and protein kinase B and further activates downstream mammalian target of rapamycin, which enhances protein synthesis.^[35–37]^

It has long been assumed that the risk factors that cause weak muscle strength in older people are closely linked to increasing age, and of course to weight loss in older people, as well as reduced muscle mass.^[38]^ As people get older, proteins (lipofuscin and cross-linking proteins) that are unable to complete contractions on their own may accumulate in skeletal muscle in older adults, reducing muscle strength, and these risk factors accelerate the onset of sarcopenia. Of course, the reduction in skeletal mass associated with sarcopenia is not necessarily accompanied by weight loss. Instead, sarcopenia usually shows no change in overall body weight, but an increase in the ratio of fat to muscle. This so-called “obesity-related sarcopenia” or “sarcopenic obesity” shows changes in muscle composition, such as “marbling,” which is caused by fat infiltrating the muscle.^[39]^ This is caused by the infiltration of fat into the muscle. In turn, can we assume that fat “marbling” leads to a decrease in muscle mass and somatic function.^[40]^ This trend is further exacerbated with age, as fat deposits shift from subcutaneous fat and intramuscular and visceral fat depots increase, and this change in muscle mass impairs muscle function and raises the patient’s risk of death.^[40]^

Insufficient or even no exercise in humans is now recognized as an important cause of sarcopenia.^[41]^ The decline in muscle fiber values in people is closely related to age, with the rate of decline being more pronounced from about age 50 onwards,^[42]^ and of course the rate of decline in muscle fibers and strength is even more pronounced in patients who are less active, or even inactive for long periods of time, compared to those who are relatively more active, even without regard to the age factor. Even professional athletes who have been inactive for a long period of time will experience varying degrees of decline in speed, endurance, and muscle strength as they age and become more inactive, though perhaps at a slower rate than the general population.^[40]^ In studies in mice, exercise has been shown to improve muscle stem cell function, promote nicotinamide adenine dinucleotide metabolism by slowing the age-related weakening of nicotinamide phosphoribosyltransferase,^[43]^ and increase the ability of aging muscles to repair damage. Therefore, active participation in sports, strengthening of the body, with a reasonable diet, maintaining a good state of mind, coupled with the nutritional support of essential amino acids can indeed improve muscle status, which is, of course, one of the most effective ways to combat sarcopenia.

### 3.4. Imbalances in neuromuscular regulation

As we age, our skeletal muscles shrink and the number of motor neurons decreases, especially in the elderly, and especially in those over 70 years of age, with the most pronounced decrease being in the alpha motor neurons. It is very likely that muscle loss occurs in humans from the age of about 30 years, and may vary with age, and the rate of loss is very likely to accelerate.^[44,45]^ According to the standards, we distinguish between slow fibers (type I) and fast fibers (type II) in human skeletal muscle fibers, and sarcopenia is characterized by the decay and contraction of type II fibers, accelerated fiber death, and a decrease in fiber cross-linking and mitochondria.^[46]^ Type II fiber atrophy, in turn, is driven by several physiological processes of aging, including loss of motor neurons, muscle denervation, instability of the neuromuscular junction, and a decrease in muscle satellite cells responsible for muscle growth, maintenance, and repair.^[4,47]^ Aging also leads to a limitation of mitochondrial function, which is characterized by a decrease in mitochondrial deoxyribonucleic acid and adenosine triphosphate production and an accumulation of intracellular reactive oxygen species in skeletal muscle.^[48]^ Atrophy of muscle fibers is accompanied by infiltration of adipose tissue, leading to loss of muscle mass.^[49]^

### 3.5. Hormonal imbalances

Growth hormone, sex hormone, and other hormones in the human body all have some degree of influence on skeletal muscle, and it has been proven and discovered that a relatively large number of growth hormone, sex hormone, insulin-like growth factor-1, and vitamin D, etc, but exactly how the influence is, and what we can do to intervene, need a lot of data to analyze and confirm. We utilize the medium of insulin-like growth factor-1 to accomplish the delivery and transfer of growth hormone, and at the same time, the medium of insulin-like growth factor-1 is also very likely to accomplish the same direction of scheduling on the serine/threonine protein kinase B channel, accelerate the process of protein formation, alleviate or even prevent the breakdown of protein links, in order to improve the muscle strength within the body, to achieve the purpose of strengthening muscle mass. mass. However, due to the different physical conditions of people, especially the elderly, their body’s growth hormone pulse secretion and release of the cycle and the range is not high, the growth hormone will also be with the age of the growing trend of a significant decline, a series of factors, resulting in the elderly relative to young people, is very susceptible to the occurrence of sarcopenia.^[50]^ Age-related changes in the endocrine system are also considered to be one of the most important functional changes, with relatively constant levels of thyroid hormones and glucocorticoids, while blood levels of sex steroid hormones, such as testosterone, are known to decrease with age in adults.^[51,52]^ A cross-sectional study found that serum testosterone levels were positively associated with skeletal muscle mass and strength in men aged 24 to 90 years. In contrast, postmenopausal women commonly experience a decline in estrogen, which may contribute to endocrine and metabolic abnormalities. These changes can lead to muscle mass loss and are strongly associated with physical frailty. This may progress to reduced muscle strength and function, increasing the risk of conditions such as osteoporosis, metabolic syndrome, and sarcopenia. However, the underlying biological mechanisms remain unclear and require further in-depth research supported by large-scale data analyses,^[53]^ but the biological mechanisms are still uncertain, but also need a large number of research data to be compared and analyzed, but also to be further in-depth research to confirm.

Multiple studies have shown that vitamin D supplementation is almost universally insufficient, especially in the elderly, and that insufficient vitamin D supplementation is one of the most important factors in the development of sarcopenia.^[1,25]^ Levels of dihydroxyvitamin D are also very likely to be strongly associated with the body’s muscle strength, muscle mass, and susceptibility to falls, among other things.^[54]^ But how does vitamin D operate in skeletal muscle cells? What channels are utilized? There is still a lot of disagreement, a lot of research to be done, and a lot of data to be confirmed.

### 3.6. Treatment of sarcopenia

#### 3.6.1. Nutritional interventions in sarcopenia

Inadequate protein supplementation can lead to nutritional imbalances and may be a risk factor for sarcopenia. Although there is still a lot of disagreement about the quantity and quality of protein supplementation, the ways and means of supplementation, as well as how to supplement other nutrients and how much multivitamin supplementation is most appropriate, most of the research results on protein supplementation for the elderly published by the European Society of Clinical Nutrition and Metabolism at the “Protein Requirements of the Elderly” Symposium in 2013 are the most important ones. However, the research results on protein supplementation in the elderly published by the European Society for Clinical Nutrition and Metabolism in 2013 at the “Protein Requirements of the Elderly” symposium are the main focus, with a special emphasis on their recommendation of a minimum protein supplementation of 1.0 g for the general population of elderly people aged 65 years and older ~1.5 g/kg-d, and > 1.5 g/kg-d for those with more severe diseases or major physical injuries.^[55]^ Currently, we believe that the best protein supplement for patients with sarcopenia is whey protein.^[56]^ At present, there are not many studies analyzing the nutrient supplementation of sarcopenia patients, especially the in-depth data exploring the nutrient supplementation of sarcopenia patients in the elderly group is even more scarce. Now, we have found from domestic and foreign research data that if people can consume a certain dose of whey protein every day, the quantity can reach 30 to 50 g, and can be consumed twice or more every day, then the muscle strength, quality and body function of patients with sarcopenia can be further strengthened, and their ability to limit their activities can be alleviated to a certain extent, and the quality of their life can be greatly improved.^[57]^

Inadequate branched-chain amino acid supplementation synergized with low activity levels and inadequate overall protein supplementation may result in a dramatic decrease in overall muscle mass.^[58]^ Branched-chain amino acids not only improve exercise endurance but also alleviate central fatigue.^[59]^ Essential amino acids can enhance both mammalian rapamycin target protein complex 1 signaling and muscle synthesis, as well as intervene in the loss of muscle strength and mass due to physical inactivity or prolonged bed rest due to illness.^[60]^ Cruz-Jentoft et al^[61]^ demonstrated that when older adults consumed leucine at a specific dosage, it not only effectively promoted skeletal muscle protein synthesis, but also enhanced muscle function. Furthermore, their findings indicated that somatic function shows significant responsiveness to leucine supplementation in the elderly population. However, if too much protein is supplemented, not only can it lead to the accumulation of body fat and other abnormalities in the body, but it can also lead to other diseases, and conversely, it is unlikely to reduce the incidence of sarcopenia. It has also been found that if after a very intense exercise, the intake of branched-chain amino acids (0.087 g/kg) in the ratio of leucine 2, isoleucine 1 and valine 1 (i.e., 2:1:1) is taken in a timely manner, it is possible to achieve the purpose of improving and enhancing muscle strength, improving muscle quality and perfecting the function of the body. However, there is no sufficient research data to confirm the specific amount of supplementation and the method of supplementation.^[62]^

Protein synthesis accelerators are composed of hydroxymethylbutyric acid, and also creatine and L-carnitine. They play a very important role in helping older people with timely protein supplementation, targeting the acceleration of the strength of the muscles to complete protein formation, and may also prevent sarcopenia from occurring to some extent. Hydroxymethylbutyric acid is a kind of leucine metabolite, which has been stored in the skeletal muscle inside the human body, and it is a kind of very useful protein synthesis enhancer in the skeletal muscle inside the human body, and it is recognized as leucine. In addition, domestic and international studies have shown that hydroxymethylbutyric acid can intervene well in sarcopenia in the elderly, and to some extent may even be able to reverse sarcopenia^[63]^ L-carnitine is an essential amino acid in the human body, and its footprints can easily be found in human skeletal muscle, and its components mainly come from the liver and kidney. L-carnitine is completely utilized to reduce the amino acid to reduce the production capacity in order to improve the formation of muscle protein. L-carnitine is utilized solely to reduce amino acid production in order to increase the formation of muscle protein. Of course, it also has the powerful ability to interfere with or even prevent the breakdown of proteins in skeletal muscle.^[64]^

A high-risk cause associated with the development of sarcopenia can also be vitamin D deficiency. Vitamin D deficiency is generally considered to be a failure of blood 25-hydroxyvitamin D levels in the range of 20.0 ng/mL, while inadequate vitamin D intake has been shown to be around 20.0 to 29.9 ng/mL. We now estimate that the average recommended dietary allowance for vitamin D is 600 IU/d in the 1 year old ~70 year old age group, but more than 800 IU/d in the 70 + year old age group. We can find vitamin D receptors in human tissue protein cells and know that it is related to the formation of muscle proteins, most likely due to the fact that vitamin D has a certain regulatory role in the synthesis of muscle proteins by using direct transcription and non-direct musculoskeletal interactions. So, 1 should also have the thinking and the belief that if a specific dose of vitamin D is taken, it can increase bone strength, improve cardiac muscle function, and can intervene in the development of sarcopenia.^[54]^ Additionally, vitamin D supplementation may also have some ameliorative effect on skeletal muscle inflammation. data from a 2019 study suggests that supplementation with vitamin D and leucine-rich whey protein, if taken for up to 13 weeks, may provide some relief from chronic inflammatory responses, particularly in older sarcopenic patients with mobility impairments.^[65]^

It has also been found that if some antioxidant and anti-inflammatory substances such as vitamin E, Astaxanthin, Omega-3 fatty acids^[66–68]^ are used in the general population, especially the elderly, not only can they improve their muscle strength, muscle mass, but also enhance mobility, thus intervening in the occurrence of sarcopenia.

#### 3.6.2. Exercise interventions for sarcopenia

Physical inactivity represents a primary etiological factor in sarcopenia development. Exercise therapy, comprising 2 principal modalities (aerobic and resistance training) constitutes the fundamental therapeutic approach for sarcopenia management.^[69]^ Aerobic exercise is the use of repeated, high-intensity activation of the body’s oversized muscle groups, so that the mitochondrial mass and the density of the capillaries play a role in the same time, resulting in the body’s internal capacity for oxygen uptake and muscular endurance have been strengthened to a certain extent.^[69]^ Resistance exercise, on the other hand, utilizes a small amount of repetitive muscle mass expansion to increase the cross-sectional area of the fibers and the strength of the muscle,^[69,70]^ thus improving muscle mass and strength. Short-term resistance exercise has been shown to enhance the ability of skeletal muscle to synthesize protein.^[71]^ According to relevant reports, resistance training can exert positive effects on the neuromuscular system, as well as enhancing hormone concentration and speeding up the rate of fusion with proteins.^[72]^ 1 published literature in 2018, the combined income of 19 clinical trials reflected that active participation in the activity, strengthening exercise, as well as a reasonable diet, and in conjunction with a targeted scientific exercise approach, can fully achieve the purpose of improving muscle strength. Can achieve the purpose of improving muscle strength, for the lower body muscle strength enhancement effect is more obvious, just for the pace, and grip strength and other aspects of the improvement of the effect is not significant.^[57]^ A team of researchers conducted a 12-week resistance exercise program to improve the mobility of elderly patients with sarcopenia and myasthenia gravis and found that the best solution to improve the condition of these patients was to supplement with both protein and vitamin D.^[73]^ The results of a recent study have revealed some benefits when using a combination of dietary supplements and exercise, but the results are not consistent across populations.^[74]^ For the time being, we can assume that the common feature of both aerobic and resistance exercise is that it should be carried out gradually and regularly, not <3 times a week for more than 30 minutes or more than 3 times a week and for no <30 minutes each time, which depending on the individual’s physical condition.^[70,75]^

#### 3.6.3. Pharmacological treatment of sarcopenia

The current therapeutic arsenal for sarcopenia remains remarkably limited in clinical practice. Dehydroepiandrosterone and human growth hormone have little effect on improving sarcopenia. Growth hormone, to a certain extent, can merely help muscles to go through the process of synthesizing proteins at an increased rate for the purpose of enhancing muscle mass, and is unlikely to lead to an increase in strength and function^[76]^; testosterone or other anabolic steroids can have some positive effects on muscle strength and mass, but may have adverse effects, and their uses are are also very limited.^[76]^ For example, cardiovascular-related risk factors are increasing in general, as well as risk factors for prostate cancer in men, especially in the older male population, and there may be a very pronounced masculinization of some women.^[77]^

Current therapeutic strategies for sarcopenia intervention continue to undergo rigorous clinical evaluation and investigation. We can consider using selective androgen receptor modulators, which have highly specific tissue targeting and are therefore widely accepted. However, currently these drugs cannot effectively use androgen signaling to increase skeletal muscle strength and mass without causing dose-related side effects.^[78]^ Other substances currently being researched for sarcopenia treatment include muscle growth inhibitors, angiotensin-converting enzyme inhibitors, vitamin D, eicosapentaenoic acid,^[76,77]^ celecoxib, thalidomide, Omega-3 supplements, and anabolic compounds like growth hormone-releasing peptides and their analogs including ruxolitinib.^[79]^ MT-102 is the first drug of its kind that can transform between anabolic and catabolic states, and has been proven in elderly animal models to increase muscle strength and mass without causing dose-related side effects. MT-102 has demonstrated the ability to reverse sarcopenia in aged animal models.^[80]^

There is a recent article that reports that if you use herbal compounds that have an effect on skeletal muscle, including alkaloids and steroidal lactones, as well as catechins, proanthocyanidins, and curcumin and gingerols.^[81]^ It can improve muscle mass and promote a speedy recovery in patients with sarcopenia,^[81]^ but research is still in its infancy and there is not a lot of supporting literature on the use of herbal compounds for diagnosing and intervening in sarcopenia, which, of course, could be one of the directions for exploring the treatment of sarcopenia in the future.

## 4. Conclusions

At present, the diagnosis of sarcopenia is difficult. Although our country has published the Chinese expert consensus on sarcopenia, it is still more based on foreign standards, and it is a subjective diagnostic threshold, which is not yet fully applicable to clinical practice. In addition, in the treatment of sarcopenia, clinical programs for elderly patients with sarcopenia are limited to the use of nutrition and exercise. In the case of nutritional therapy, more tests and data are needed to determine the usage and dosage of nutritional supplements, whether the patients are adapted to them, whether there are any adverse effects, and whether the therapeutic goals are achieved.

Strengthening the management of patients with sarcopenia has a very positive significance on improving the nutritional status and prognosis of hospitalized patients, and should attract enough attention and intervention.

## Author contributions

**Conceptualization:** Hongjun Yang.

**Data curation:** Jianbin Zhang.

**Investigation:** Jianbin Zhang.

**Visualization:** Hongjun Yang.

**Writing – original draft:** Xuejiao Xian.

**Writing – review & editing:** Hongjun Yang.
